# Supplementary material for: Machine learning based refined differential gene expression analysis of pediatric sepsis
Source: BMC Med Genomics. 2020 Aug 28;13:122. doi: 10.1186/s12920-020-00771-4 (PMC7453705; doi:10.1186/s12920-020-00771-4)
Supplement: Supplementary file 2 — Additional file 2. Supplementary Figs. S1-S4. [file 12920_2020_771_MOESM2_ESM.pdf]

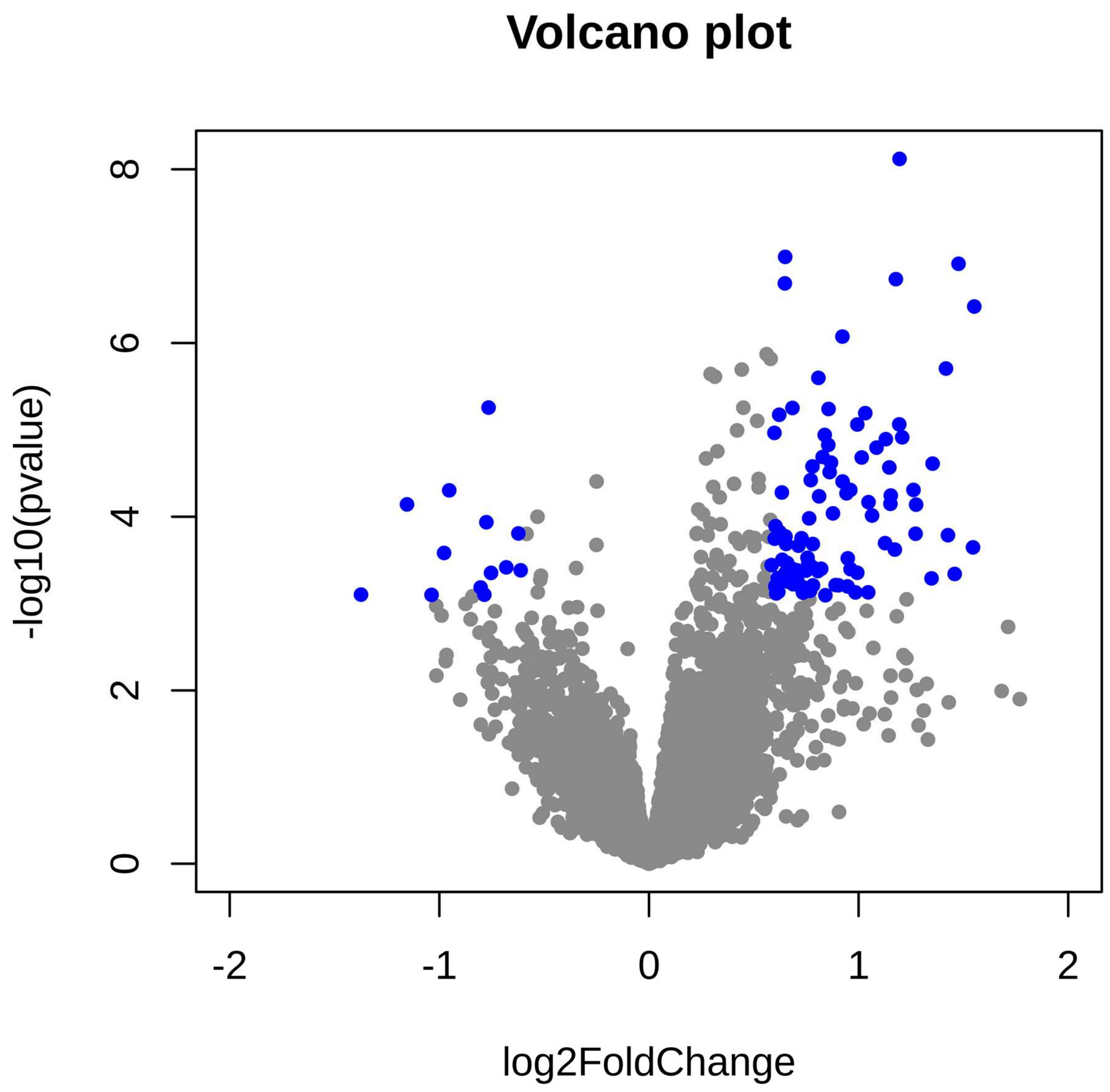

Fig. S1 Volcano plot of gene expression genes in pediatric sepsis. Genes highlighted in blue represent significantly up-regulated (right) and down-regulated (left) genes.

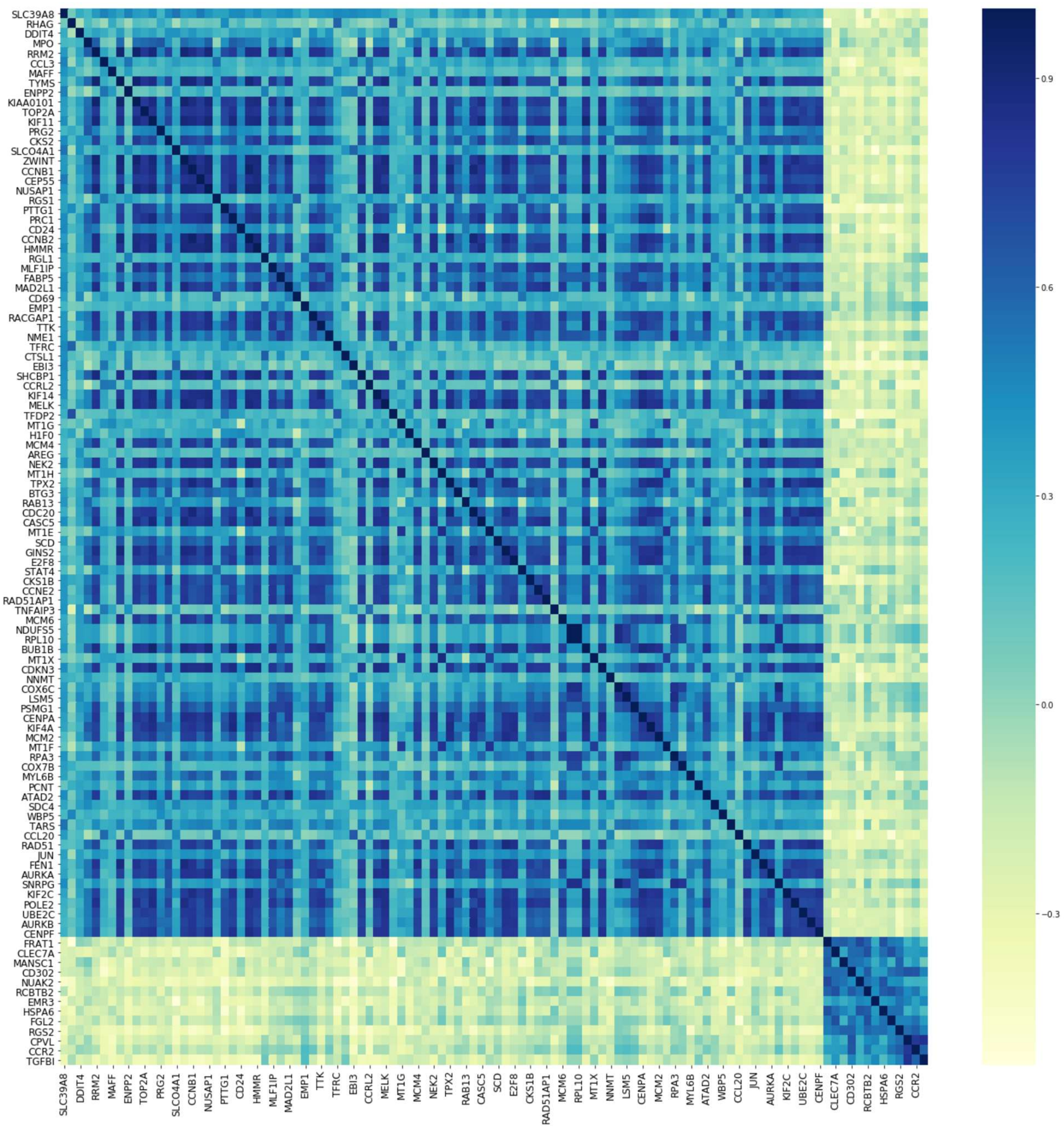

Fig. S2 Heatmap of Pearson's correlation coefficient matrix of the 108 DEGs.

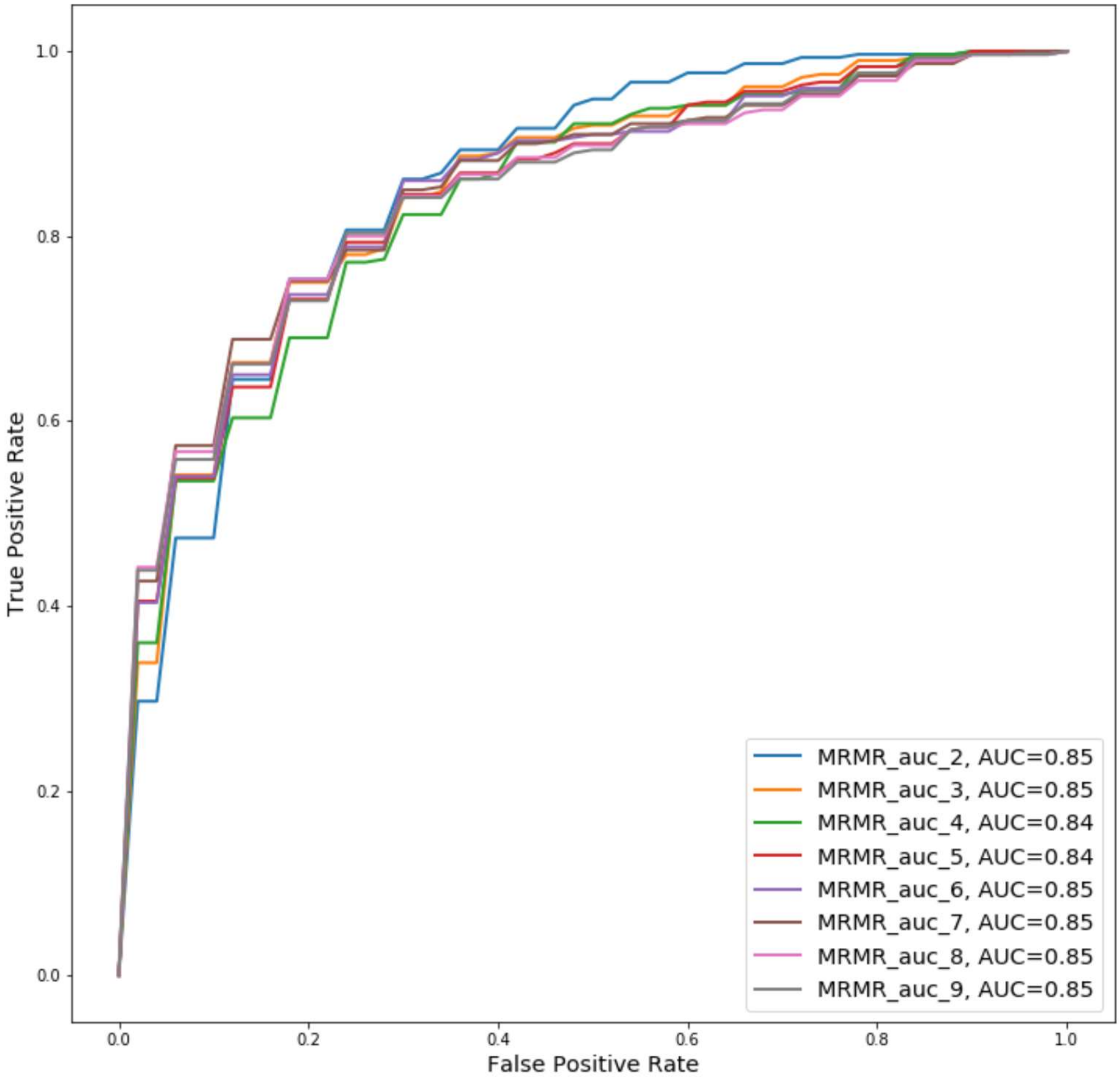

Fig. S3 Average ROC curves of LR models estimated using 10 runs of 10-fold cross-validation and MRMR_auc for selecting top 2,3,…,9 marker genes from the 108 DEGs.

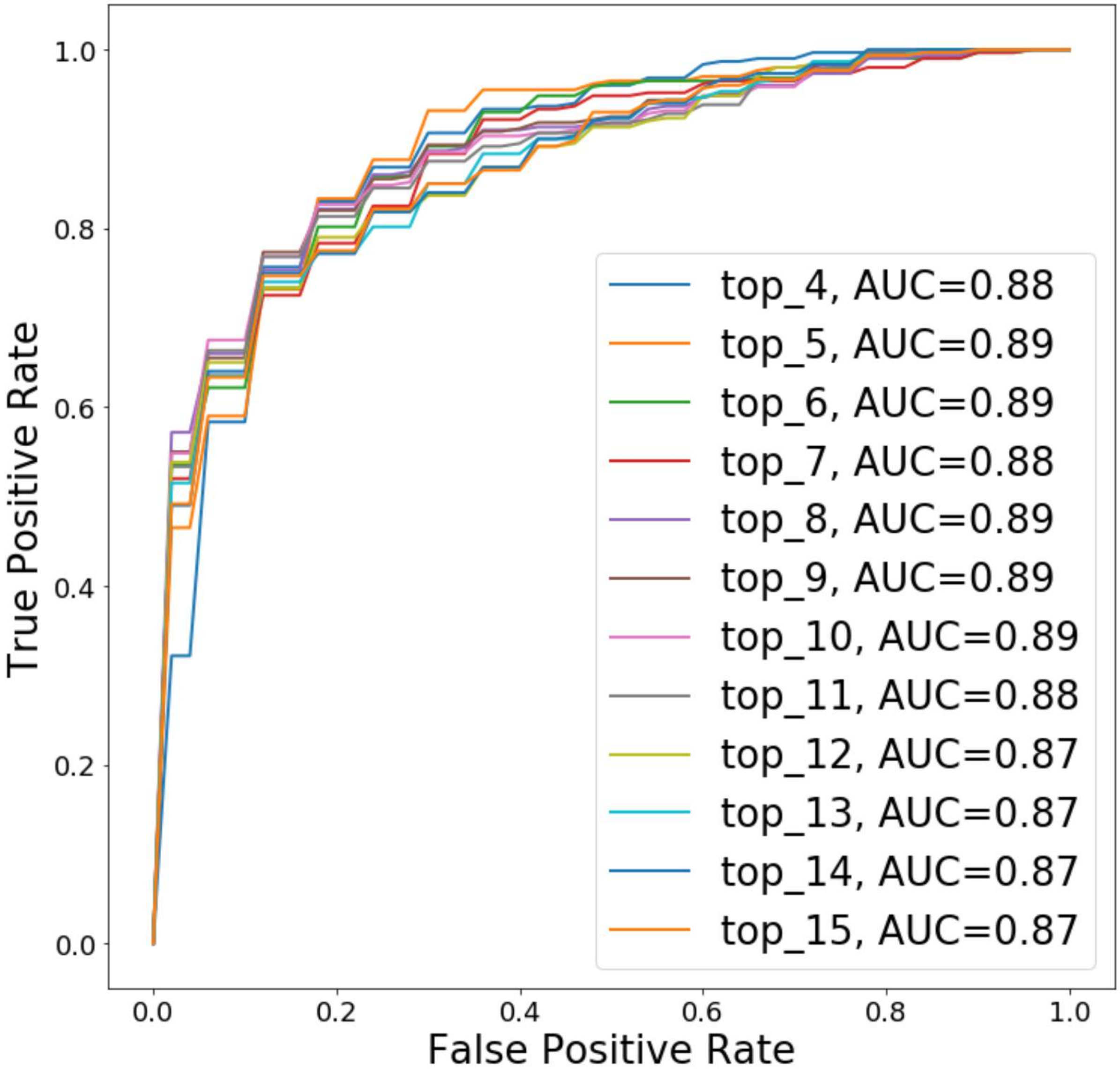

Fig. S4 Average ROC curves of LR models estimated using 10 runs of 10-fold cross-validation and top re-ranked 4,5,…15 marker genes.
